# Giant seminal vesicle cyst with hemorrhage in Zinner syndrome: A case report

**DOI:** 10.1097/MD.0000000000031577

**Published:** 2022-12-16

**Authors:** Qingfei Xing, Li He, Xinghua Gao, Chunling Zhang, Xudong Luo, Xiangqin Gao, Yanlin Wu, Longyang Zhang, Feng Guo

**Affiliations:** a Department of Urology, Central Hospital Affiliated to Shandong First Medical University, Jinan, Shandong Province, PR China; b Department of Health, Shandong Province Hospital, Jinan, Shandong Province, PR China; c Department of Medical Imaging, Central Hospital Affiliated to Shandong First Medical University, Jinan, Shandong Province, PR China.

**Keywords:** case report, ipsilateral renal agenesis, seminal vesicle cysts, Zinner syndrome

## Abstract

Zinner syndrome (ZS) is a rare congenital malformation of the urogenital tract that is associated with seminal vesicle cysts, ejaculatory duct obstruction, and ipsilateral renal agenesis. This unique condition was first reported by Zinner (1914). ZS is caused by malformation of the distal mesonephric duct during embryogenesis. To our knowledge, no giant seminal vesicle cysts with hemorrhage in ZS have been reported in the current study.

**Patient concerns:**

A 63-year-old man presented with chronic hypogastralgia with no history of lower urinary tract symptoms, hematuria, or trauma. Physical examination revealed no localized uplift or percussive pain in either kidney. No tenderness in the ureter stroke region, no localized eminence in the suprapubic region of the bladder, and no tenderness in the bladder region was observed. Digital rectal examination revealed a cystic mass with a smooth surface in the anterior wall of the rectum with no tenderness or unclear boundaries. No blood staining was observed in the finger sheaths.

**Diagnoses:**

Computed tomography scan revealed that the right kidney was absent, with a mass similar to a cord above the right seminal vesicle cyst. Contrast-enhanced pelvic magnetic resonance imaging (MRI) confirmed a short T1 and T2 signal shadow similar to a cord above the right seminal vesicle cyst. The boundary was clear, with the upper part leading to the “renal region” and the lower part connecting to the right seminal vesicle cyst. Contrast-enhanced MRI showed local parenchymal cysts with cyst wall enhancement but no intrathecal enhancement. This suggested a hemorrhagic cyst. A diagnosis of Zinner syndrome was established.

**Interventions:**

The patient was diagnosed with a giant seminal vesicle cyst with hemorrhage in ZS. The patient had no obvious symptoms; therefore, regular follow-ups were performed.

**Outcomes:**

MRI of the patient 1 month later showed that the hematoma in the seminal vesicle cyst was not absorbed.

**Lessons:**

Giant seminal vesicle cysts with hemorrhage in ZS are rare. To patients without symptom, regular follow-up can be adopted.

## 1. Introduction

Zinner syndrome (ZS) is a rare congenital malformation, which was first reported by Zinner in 1914, and is characterized by seminal vesicle cysts, ejaculatory duct obstruction, and ipsilateral renal agenesis.^[1]^ The frequency of this condition is reported to be 0.0046% according to Farooqui et al.^[2]^ The majority of patients with ZS have no obvious clinical symptoms. Common symptoms include dysuria, frequency, perineal pain, hematuria hemospermia and didymitis. The diagnosis depends on color ultrasound (US), computed tomography (CT), and magnetic resonance imaging (MRI) scans, but MRI has a better ability to identify soft tissue and provides a complete image of the local anatomy.^[3]^ Management of seminal vesicles cysts depends on the presentation and presence of symptoms, and conservative treatment is often used in asymptomatic patients. Malignant cysts with significant clinical symptoms and a cyst with a diameter of ≥2.5 cm are considered for surgical treatment.^[4]^ Minimally invasive surgeries include the laparoscopic, robotic-assisted approaches, simple cyst drainage or transrectal aspiration.

## 2. Case report

**Figure 1.**
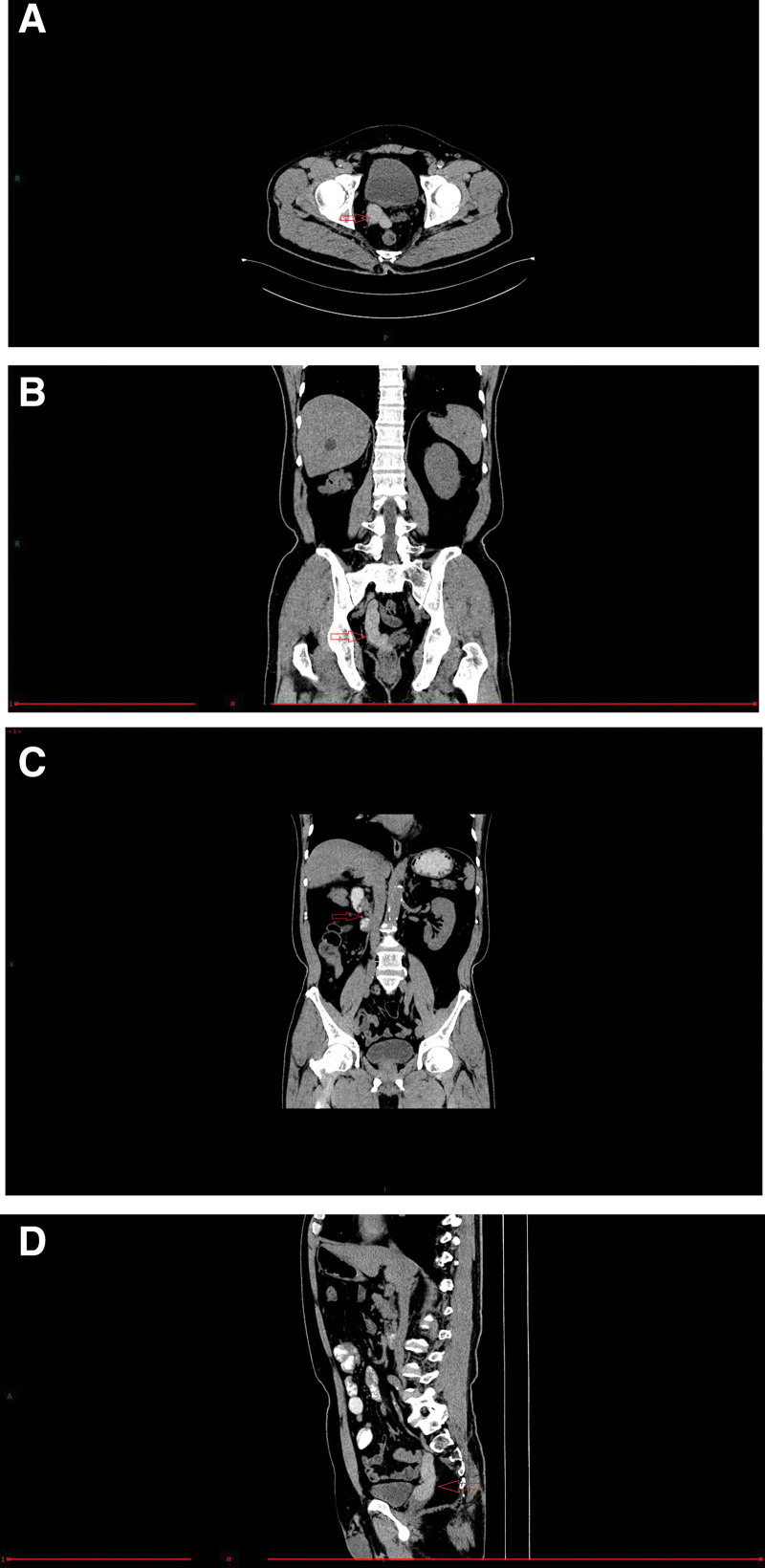
Computed tomography (CT) demonstrates a cord-like mass (arrow) above the right seminal vesicle cyst; (A, B, C) the boundary was clear, with the upper part leading to the “renal region” and the lower part connecting to the right seminal vesicle cyst, and (D) the absence of the right kidney (arrow).

**Figure 2.**
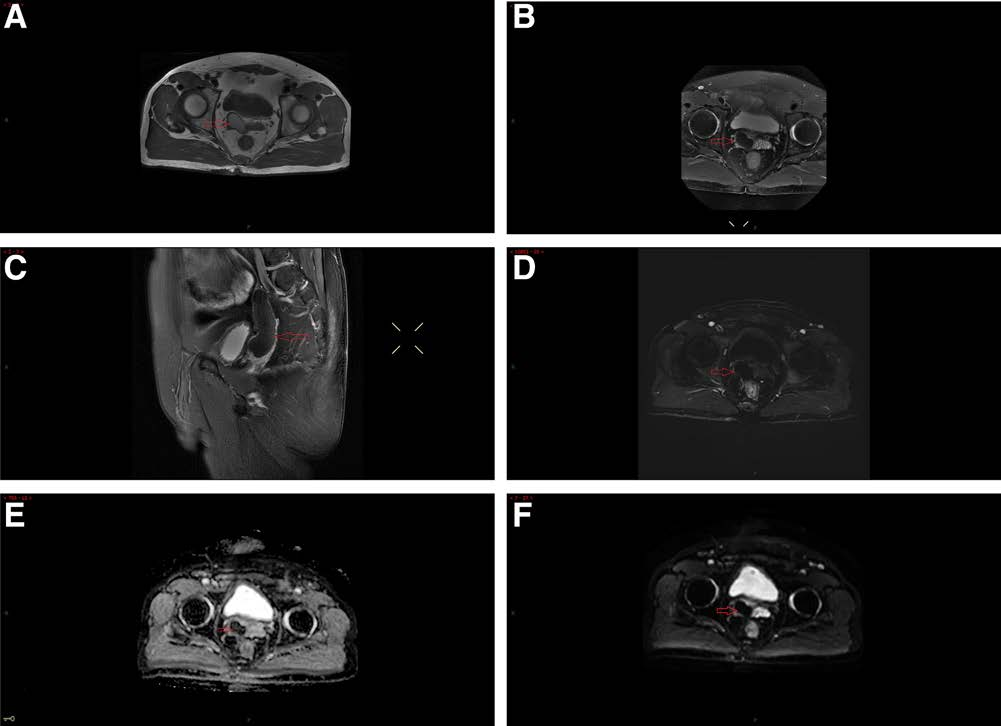
Contrast-enhanced pelvic magnetic resonance imaging (MRI) confirmed a (A) short T1, (B) T2, and (C) signal shadow(arrow), similar to a cord above the right seminal vesicle cyst(arrow). The boundary was clear, with the upper part leading to the “renal region” and the lower part connecting to the right seminal vesicle cyst (arrow). (D) Contrast-enhanced MRI revealed local parenchymal cysts with cyst wall enhancement, but no intrathecal enhancement (arrow). (E) ADC showed low signal intensity (arrow), (F) DWI showed low signal intensity (arrow).

**Figure 3.**
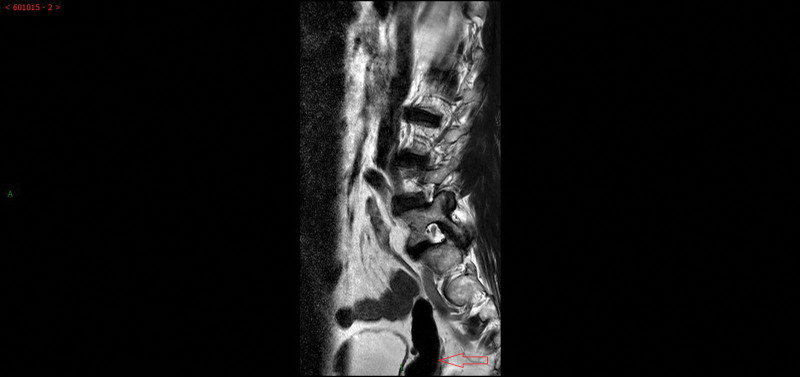
Lumbar MR examination showed that the hematoma in the seminal vesicle cyst (arrow) was not absorbed.

A 63-year-old man without any relevant medical history was admitted to the hospital due to “hypogastralgia with discomfort in the perineal area for 4 weeks.” The patient had no obvious dysuria, hematuria, urination frequency, urgency, or waist pain. He was married at the right age, and had a daughter. Physical examination showed no localized uplift and no percussive pain in either kidneys. No tenderness in the ureter stroke region, no localized eminence in the suprapubic region of the bladder, and no tenderness in the bladder region was observed. The external genitalia were normal, and normal testes and epididymis were palpable on both sides of the scrotum. Digital rectal examination revealed a cystic mass with a smooth surface in the anterior wall of the rectum with no tenderness or unclear boundaries. No blood staining was observed in the finger sheath. Laboratory test results revealed no abnormalities. No obvious abnormalities were observed in prostate-specific antigens. In the absence of the right kidney and a cord-like mass above the right seminal vesicle cyst, the CT value was approximately 70 HU, and the boundary was clear, with the upper part leading to the “renal region” and the lower part connecting to the right seminal vesicle cyst (Fig. 1A–D). Contrast-enhanced pelvic MR confirmed a short T1 and T2 signal shadow, similar to a cord above the right seminal vesicle cyst. The boundary was clear, with the upper part leading to the “renal region” and the lower part connecting to the right seminal vesicle cyst. The ADC and DWI showed low signal intensity. No lymphadenopathy was observed in the iliac or pelvic effusions. Contrast-enhanced MRI revealed local parenchymal cysts with cyst wall enhancement, but no intrathecal enhancement, suggesting a hemorrhagic cyst (Fig. 2A–F). A diagnosis of Zinner syndrome was established. The patient had no obvious symptoms; therefore, regular follow-ups were performed. Lumbar MR examination of the patient 1 month later, due to lumbar pain caused by trauma, showed that the hematoma in the seminal vesicle cyst was not absorbed (Fig. 3).

## 3. Discussion

ZS is a rare congenital anomaly of the urogenital tract that is usually discovered and diagnosed in the 2nd to 4th decade of life.^[5]^ It is difficult to estimate the prevalence correctly. According to an investigation that 2,80,000 children over 2 and a ½ years of age underwent ultrasonographic screening in Taiwan in 1990 and the prevalence of ZS was 0.00464%.^[6]^ Chinese researchers discovered 214 cases by retrieving the publications on ZS between 1999 and 2020. However, to our knowledge, no giant seminal vesicle cyst with hemorrhage in ZS was reported in the current studies. However, the exact cause of this syndrome remains unknown. The urinary and male reproductive systems both arise from the mesonephric duct. Under normal circumstances, starting from the 5th week of embryo development, the end of the mesonephric duct under the influence of male hormones and anti-Muller hormones can give rise to vas deferens, ejaculatory tubes, epididymis and other reproductive ducts,^[7]^ and the end near the cloaca can also send out a blind tube to the dorlateral side, which is called the ureter bud, and the latter will gradually differentiate into ureter and renal collection systems and stimulate the development of the buds of the metanephritis to form the metanephritis. Congenital dysplasia of the mesonephric duct may inhibit the interaction between the ureteral bud and the metanephron, ultimately resulting in renal and ureteral hypoplasia. Failure of the ureteral bud to separate from the distal end of the mesonephric duct leads to ejaculatory duct atresia.^[8]^ Some ureteral traces may remain in the seminal vesicle, which leads to duct obstruction or atresia. A cyst is created or formed when insufficient drainage secretions in the gland are lined or related to atresia, which later causes distention of the seminal vesicle.^[9]^

Most patients with this syndrome are usually asymptomatic, but can present with nonspecific symptoms such as dysuria, frequency, perineal pain, hemospermia, hematuria or epididymitis.

Many imaging techniques have been used to evaluate and discriminate between pelvic cystic masses. As a result, radiological modalities including US, CT, and MRI play a significant role in diagnosing and evaluating ZS. US can be used as the first choice of examination, including abdominal and transrectal US. Abdominal ultrasonography revealed the cystic nature of the mass, its size and location, and the absence of the ipsilateral kidney. Transrectal ultrasonography provides more information about the cyst wall and its contents. A CT and MRI can be used as secondary diagnostic tools. CT findings might include ipsilateral kidney agenesis in addition to a retrovesicular periprostatic cystic mass. MRI is considered the imaging modality of choice for diagnosing this condition owing to its high-resolution properties.^[5]^ CT and MRI can not only show the relationship between the seminal vesicle cyst and the prostate and rectum more clearly but also classify the seminal vesicle mass as a solid or cystic mass.^[10]^

MRI has better recognition ability for soft tissue to determine the presence of hemorrhage inside the cyst and can also differentiate other pelvic cystic masses, such as prostatic cyst, Muller cysts, ejaculatory duct cysts, and seminal vesicle tumors, etc.^[11]^ MRI showed low intensity on T1-weighted images and high intensity on T2-weighted images in case of seminal vesicle cysts. Imaging showed increased intensity on T1-weighted images in cases of intracranial hemorrhage or infection.^[12]^

The treatment of the seminal vesicle cysts was unified. Asymptomatic patients diagnosed with seminal vesicle cysts can remain under observation unless malignancy is suspected. Surgical treatment was adopted for patients with malignant cysts, obvious clinical symptoms, and cysts with a diameter of >2.5 cm.^[13]^ If patients are with infertility, surgical treatment should be adopted to improve fertility.^[14]^ Different management strategies have been reported in the literature. Surgical methods include transrectal and perineal seminal vesicle cysts puncture and drainage, open seminal vesicle cystectomy, laparoscopic and robot-assisted seminal vesicle cystectomy, etc.^[2,15,16]^ Because of the deep location of the seminal vesicles in the retrovesical space, traditional open surgery field exposure is poor, and it has significant trauma, slow recovery, and postoperative complications. Therefore, open surgical access is technically challenging. Laparoscopic surgery is characterized by good surgical field exposure, small trauma, quick recovery, fewer postoperative complications, and high safety.^[17]^ In recent years, urology departments have performed laparoscopic radical resection of prostate cancer and bladder cancer. A surgeon accumulates a lot of experience, and can reduce intraoperative damage to the rectum, bladder, vas deferens, and ureters. Laparoscopic seminal vesicle cystectomy is considered the safest and most effective treatment method. Furthermore, the robot-assisted approach offers enhanced dexterity and better precision.

The issue of fertility in patients presenting with symptomatic seminal vesicle cysts has mostly been overlooked. With the gradual maturity of urethral endoscopy, more reports on the treatment of seminal vesicle cysts by urethral endoscopy have been conducted, but result in a decreased success rate in comparison to open or minimally invasive surgery.^[18,19]^ Other techniques such as transrectal and perineal seminal vesicle cyst puncture and drainage may relieve patient symptoms; however, with an increased risk of recurrence, return of symptoms, and possible infection. As a result, long-term follow-up is needed to further verify the postoperative efficacy and complications.

ZS is a rare, symptomatic malformation disorder. Treatment depends on clinical signs. Surgical intervention is the mainstay of management for symptomatic patients. Laparoscopic treatment is an effective and safe surgical method for the treatment of seminal vesicle cysts. To patients without symptom, regular follow-up can be adopted.

## Author contributions

**Conceptualization:** Qingfei Xing, Xiangqin Gao.

**Formal analysis:** Xinghua Gao, Yanlin Wu.

**Image recognition:** Xudong Luo, Chunling Zhang.

**Software:** Chunling Zhang, Xudong Luo.

**Writing—original draft:** Qingfei Xing, and Li He.

**Writing—review and editing:** Longyang Zhang, Feng Guo.
